# Therapeutic Effects of a WeChat Mini-Program on Metabolic Dysfunction–Associated Fatty Liver Disease: Randomized Controlled Trial

**DOI:** 10.2196/76204

**Published:** 2026-01-27

**Authors:** Chao Sun, Guangyu Chen, Cuicui Shi, Haixia Cao, Ruixu Yang, Jing Zeng, Xiaoyan Duan, Xin Sun, Jian-Gao Fan

**Affiliations:** 1 Center for Fatty Liver Disease, Department of Gastroenterology, Xinhua Hospital, Shanghai Jiao Tong University School of Medicine, 1665 Kong Jiang Road, Shanghai, 200092, China, 86 02125077340; 2 Clinical Research and Innovation Unit, Xinhua Hospital, Shanghai Jiao Tong University School of Medicine, Shanghai, China

**Keywords:** metabolic dysfunction-associated fatty liver disease, WeChat mini-program, lifestyle intervention, weight loss, metabolic parameters, randomized controlled trial

## Abstract

**Background:**

For patients with metabolic dysfunction–associated fatty liver disease (MAFLD), weight loss is advised but challenging in practice. In China, there is a pronounced shortage of tailored digital lifestyle interventions for this population.

**Objective:**

This study aimed to assess the effects of a WeChat mini-program-delivered lifestyle intervention on weight loss and hepatic steatosis among individuals with MAFLD who were overweight or obese.

**Methods:**

Adults who are overweight or obese and have clinically diagnosed MAFLD with transient elastography examination were enrolled in this prospective randomized controlled trial. Patients were randomly assigned to receive either WeChat mini-program management (intervention group) or standard care (control group) at a 1:1 ratio. The intervention was structured around the development and implementation of personalized diet and exercise plans, supplemented by guided exercise video courses and reinforced through continuous monitoring and informational support. Body weight and clinical parameters were assessed at baseline and then at 6 months.

**Results:**

A total of 89 patients met the inclusion criteria and were randomly assigned to the intervention group (n=45) or control group (n=44). Among the 89 patients with MAFLD, 60% (27/45) of them achieved a weight loss of ≥5%, and 24.4% (11/45) of them had a weight loss of ≥10% in the intervention group, which was greater than those in the control group (27/45 vs 7/44; relative risk [RR] 3.771, 95% CI 1.836‐7.748; *P*<.001; 11/45 vs 3/44, RR 3.585, 95% CI 1.072‐11.988; *P*=.02). Importantly, patients receiving the intervention were significantly more likely to achieve a ≥10% reduction or normalization of controlled attenuation parameter (CAP) than those in the control group (26/45 vs 14/44; RR 1.816, 95% CI 1.102‐2.992; *P*=.01). After adjusting for key baseline covariates, multivariate analysis confirmed the intervention’s positive effect on achieving a weight loss of ≥5% (OR [odds ratio] 8.380, 95% CI 2.886‐24.331; *P*<.001) of ≥10% (OR 4.612, 95% CI 1.138‐18.686; *P*=.03), as well as on CAP reduction of ≥10 % or normalization (OR 2.853, 95% CI 1.092‐7.456; *P*=.03). In parallel, the intervention group presented greater reductions in liver enzymes (alanine aminotransferase, aspartate aminotransferase, and γ-glutamyl transpeptidase) and metabolic parameters (fasting insulin, hemoglobin A_1c_, and triglyceride) than the control group (all *P*<.05). According to the fibrosis assessment, only the FibroScan-aspartate aminotransferase score decreased more in the intervention group than in the control group (median difference −0.06, 95% CI −0.13 to −0.01; *P*=.02), as compared to other non-invasive indicators.

**Conclusions:**

Readily scalable in primary care and varied-resource settings, our WeChat mini-program-based intervention extends beyond weight loss to reduce hepatic steatosis and improve metabolic parameters, thereby addressing the critical gap in targeted MAFLD management in China with a low-cost model for high-burden populations. Nevertheless, larger future studies are needed to confirm these findings with greater precision and assess long-term sustainability.

## Introduction

### Background

Metabolic dysfunction–associated fatty liver disease (MAFLD) represents a significant and growing public health burden worldwide, paralleling the epidemics of obesity and type 2 diabetes mellitus [1]. It is a gradually progressive disease that evolves through a spectrum that begins with simple hepatic steatosis and advances to metabolic dysfunction-associated steatohepatitis (MASH), ultimately resulting in liver fibrosis, cirrhosis, and even hepatocellular carcinoma [1,2]. Despite its severe clinical sequelae, there are no pharmacologic therapies approved in China to reverse the histological progression of MAFLD, underscoring the critical role of nonpharmacological management [3].

Lifestyle intervention involving dietary adjustments and physical exercise is still the cornerstone of MAFLD management. Clinical guidelines confirm that a body weight reduction of 3%-5% can ameliorate hepatic steatosis, a 7%-10% loss can improve MASH, and over 10% may even lead to fibrosis regression [3]. However, the real-world implementation of these interventions faces significant challenges [4,5].

Traditional lifestyle interventions rely on face-to-face guidance from clinical doctors in hospital settings, which often results in poor execution and low compliance, limiting the coverage and sustainability of treatment and seriously affecting patient outcomes [6]. A cross-sectional survey from China revealed suboptimal self-management of lifestyle behaviors among adults with MAFLD [7]. Lack of motivation and real-time supervision are key contributing factors to the inadequate effectiveness of lifestyle interventions. In view of this, there is an urgent need for more convenient and effective measures to enhance the implementation and adherence of lifestyle changes for patients with MAFLD, thereby improving the prognosis.

With the popularization of the internet and the rapid development of artificial intelligence, digital therapeutics (DTx), such as mobile health apps or programs, have emerged as a promising solution to bridge this care gap [8,9]. By delivering personalized health information and professional guidance, DTx enables convenient, home-based self-management, which can correct unhealthy lifestyle habits and improve treatment adherence [10-12]. A meta-analysis of 8 studies demonstrated that DTx interventions lasting 4‐24 months achieved a clinically significant weight loss at a rate of 33%, accompanied by improvements in liver enzymes and reduced liver fat [13]. In China, the near-ubiquitous use of WeChat offers a unique platform for such interventions [14,15]. Its widespread adoption eliminates the need for additional app downloads, fosters user trust through a familiar interface, and facilitates seamless communication between health care providers and patients, creating an ideal ecosystem for closed-loop chronic disease management [16,17].

Despite this potential, there is a notable scarcity of WeChat mini-programs specifically designed and rigorously evaluated for structured lifestyle intervention in Chinese patients with MAFLD. To address this gap, we developed an innovative interactive WeChat mini-program, named the therapeutic lifestyle changes (TLCs) program, which is designed to assist patients in implementing sustainable lifestyle changes for the management of MAFLD. The program offers a suite of features, including weight loss goal setting, diet records and recommendations, and instructional exercise videos.

### Objectives

Therefore, the aim of this study was to evaluate the efficacy of this novel TLC program in managing MAFLD. We hypothesized that, compared to participants receiving standard care, those engaged in the TLC intervention would demonstrate significantly greater improvements in reduction of liver fat content and body weight, as well as glucose and lipid metabolic parameters.

## Methods

### Study Design and Setting

A 6-month parallel, randomized controlled trial was conducted at Xinhua Hospital, affiliated with Shanghai Jiao Tong University School of Medicine, a tertiary-care hospital in China. The study period for participant recruitment and data collection spanned from August 2022 to May 2024 at our hospital. All eligible participants were randomly assigned to either the TLC program management group (intervention group) or the standard care group (control group) at a 1:1 ratio. The protocol (which was retrospectively registered) with no deviations was approved by the Institutional Review Board (IRB) prospectively. The reporting of this trial adhered to the CONSORT (Consolidated Standards of Reporting Trials) statement [18], with the checklist presented in Checklist 1.

### Participants

The participants with clinically diagnosed MAFLD were consecutively recruited by clinicians from our hepatology clinic. Those who were interested provided written informed consent. All participants underwent an initial, face-to-face eligibility assessment at Xinhua Hospital according to the study criteria. The inclusion criteria were as follows: (1) aged 18‐65 years, (2) BMI between 24 and 35 kg/m^2^, (3) clinically diagnosed with MAFLD by radiological features with ultrasound confirmation and a controlled attenuation parameter (CAP) value of ≥248 dB/m, and (4) able to learn and operate a mobile-based program. Patients were excluded if they had excessive alcohol consumption (weekly ethanol intake of ≥210 g for males and ≥140 g for females weekly), etiological evidence of an alternative liver disease, such as chronic viral hepatitis, autoimmune liver diseases, drug-induced hepatic steatosis, hepatic decompensation, severe comorbidities (severe cardiopulmonary disease, uncontrolled diabetes, chronic kidney disease, psychiatric disease, and malignant tumors), recent weight loss, or weight loss medication intake.

### Randomization

After the completion of baseline assessments, eligible participants were registered in the study. They were then automatically and randomly allocated to each study group at a 1:1 ratio by a computer-generated random system. The allocation was concealed until the moment of assignment, as the outcome was only revealed by the system after the participant’s formal registration. Given the nature of the digital intervention, participants and health care providers were not blinded to group assignment, but outcome assessors were blinded during data analysis.

### Lifestyle Intervention

#### “Design of the TLC program”

The TLC program-based lifestyle intervention was collaboratively designed by physicians from various departments, including Gastroenterology, Sport Rehabilitation, and Clinical Nutrition. Individuals in the control group were provided with standard care in the clinic according to the established treatment guidelines [19]. The TLC program intervention spanned 24 weeks and was divided into 4 distinct modules, which are described below.

#### Development of Diet and Exercise Plans

Individualized weight loss and exercise goals were set based on the baseline body weight, existing metabolic disorders, and severity of fatty liver disease. The target for weight reduction was established as 5%-10% of the initial body weight.

#### Dietary Intervention Plan

Based on preset weight loss goals, the program calculated daily dietary energy requirements, providing a recommended meal plan with the corresponding energy intake. If patients did not prefer the recipes suggested by the program, they could use the food exchange list to select alternative food with equivalent energy values. The program required patients to log and upload photos of their daily food intake, after which a human nutritionist analyzed the diet log and provided feedback via WeChat.

#### Exercise Video Courses

Participants were provided with a heart rate armband (HW702, YESOUL) to track heart rate during exercise. During each exercise session, data regarding heart rate were automatically uploaded by the armband. Each course lasted 30 minutes, and patients were to choose the training course at least 5 times per week. Additionally, patients could select other forms of exercise and record the duration of exercise, such as fast walking, running, cycling, and swimming.

#### Monitoring and Information Support

Patients were expected to complete their diet and exercise tasks through a check-in system every day. The research team sent tailored feedback messages weekly according to the status of task completion and offered regular suggestions through WeChat. Moreover, the system intelligently provided relevant educational articles, which provided professional information and support tailored to the patient’s interests and concerns.

### Measurements

#### Clinical Characteristics

Clinical characteristics regarding anthropometric and laboratory data were obtained at our hospital using a standard protocol at baseline and 6 months. Body composition was assessed via a bioimpedance analyzer (MC-980MA, TANITA). CAP and liver stiffness measurement (LSM) were performed using the FibroScan-502 device (Echosens) with an M or XL probe, following the manufacturer’s guidelines. A reliable examination was defined as at least 10 valid measurements and an IQR to median ratio of LSM below 30%. Based on an individual patient data meta-analysis, CAP thresholds of 268 and 280 dB/m were identified as optimal cutoffs for diagnosing moderate and severe steatosis, respectively [20].

#### Dietary Nutrition and Physical Activity Assessment

Dietary nutrition was assessed through a face-to-face interview with a questionnaire developed by the Chinese Society of Health Management and the Chinese Nutrition Society, which consists of 24 questions with a total score of 100 [21]. A score below 60 indicates a risk of dietary nutrition issues; a score between 60 and 75 suggests a potential risk; and a score above 75 indicates no dietary nutritional risk. Physical activity levels were evaluated via the International Physical Activity Questionnaire-Short Form (IPAQ-SF), which captures data on activity intensity, frequency, and daily duration in a typical week. Energy expenditure was quantified by the metabolic equivalent of task (MET) [22]. The weekly physical activity level for a specific intensity was calculated as the MET value of the activity× weekly frequency (d/wk)× daily duration (min/d).

#### Diagnostic Criteria

Obesity was considered a BMI≥28 kg/m^2^ [3]. The definition of MetS adhered to previously published criteria [23]. The homeostasis model assessment-insulin resistance (HOMA-IR) score was calculated as fasting insulin (mU/L)× fasting plasma glucose (FPG, mmol/L)/22.5. Insulin resistance was characterized by a HOMA-IR score of 2.5 or higher [1]. The fatty liver index was computed to indirectly assess the degree of hepatic steatosis using an established formula [24]. The FibroScan-aspartate aminotransferase (FAST) score was used to identify patients with MASH with significant fibrosis [25], whereas the aspartate aminotransferase-to-platelet ratio index (APRI), fibrosis-4 (FIB-4) index, nonalcoholic fatty liver disease fibrosis score (NFS), and Agile 3+ score were used to assess the risk of advanced fibrosis [26-29].

#### Outcome Measurements

The analyses included all participants who were randomized. The intention-to-treat (ITT) population for efficacy evaluation in this trial consisted of all cases that were randomized and had efficacy baseline records. The per-protocol (PP) population included participants who completed the entire planned observation period and had no major protocol deviations. The primary efficacy endpoint was the reduction of body weight by at least 5%. The secondary endpoints included a CAP value of ≥10% reduction or normalization and changes in the levels of liver enzymes and lipid and glucose parameters.

### Sample Size Calculation

The sample size calculation was based on the primary study outcome, which is the proportion of patients achieving a weight loss of ≥5% from baseline. It was postulated that the intervention would produce a 5-fold increase in the success rate from a baseline of 8% in the control group [30]. To detect this effect with 80% power at a 5% significance level and accounting for a 20% dropout rate, a total sample size of 84 participants was required.

### Data Analysis

Statistical analyses were performed using SPSS 23.0 software (IBM Corp). Missing data were due to patient dropouts and imputed by the last observation carried forward. Regarding the primary endpoint, this approach was chosen as it provides a conservative estimate of the treatment effect by assuming no further improvement in their weight status due to the discontinuation of active intervention, thereby avoiding overoptimistic extrapolation [6,31,32]. Descriptive statistics were conducted for all variables, with means (95% CIs) or medians (IQRs) reported for continuous variables and percentages for categorical variables. Categorical variables were analyzed using the chi-square test or Fisher exact test between groups (reporting relative risk and 95% CI) and the marginal homogeneity test within each group. Continuous variables were assessed using 2-tailed paired *t* tests or Wilcoxon signed-rank tests within each group, whereas unpaired *t* tests (reporting mean difference and 95% CI) or Mann-Whitney *U* tests (reporting Hodges-Lehmann median difference and 95% CI) were used to compare the changes from the baseline between groups. Binary logistic regression was used to evaluate the effect of the intervention on weight and CAP changes. The results are presented as odds ratios (ORs) with 95% CIs. A 2-tailed *P* value of .05 was considered significant.

### Ethical Considerations

This research was approved by the IRB of Xinhua Hospital (XHEC-C-2021-076-2) and was retrospectively registered with the Chinese Clinical Trial Registry (ChiCTR2500100197). This trial was registered retrospectively owing to disruptions in the research team’s workflow and uncertainties about the study’s continuity during the COVID-19 pandemic in China. We confirm that all data collection for this study was initiated subsequent to receiving approval from the IRB of Xinhua Hospital, with the IRB approval document provided in Multimedia Appendix 1. The study start was delayed due to COVID-19, and there were no other deviations or changes from the protocol after IRB approval and trial commencement. All eligible participants provided written informed consent. Participants were informed of their voluntary participation and their right to withdraw from the study at any time. It was clarified that all study procedures were offered at no cost, but no financial compensation would be made upon study completion. All personally identifiable information was coded with a unique study ID, and the anonymized data were securely stored in a password-protected cabinet.

## Results

### Participants and Baseline Characteristics

**Figure 1.**
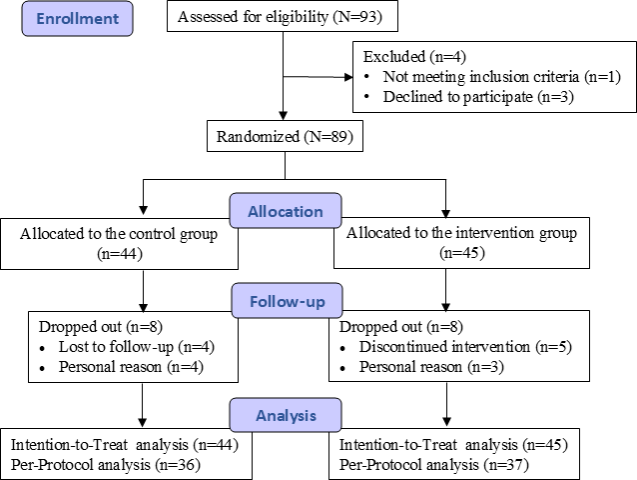
CONSORT (Consolidated Standards of Reporting Trials) flow diagram.

Figure 1 presents the study flowchart in accordance with the CONSORT guidelines. A total of 89 patients met the inclusion criteria and were randomly assigned to the intervention group (n=45) or control group (n=44); these patients comprised the ITT population. At the 6-month follow-up point, 8 participants were lost to follow-up in the intervention group and 8 in the control group. A total of 73/89 (82%) patients successfully completed the study and comprised the PP population, while the remaining 16/89 (18%) patients dropped out (Figure 1). The baseline characteristics of the patients are shown in Table 1. The mean age of the whole study population was 40.0 (SD 9.8) years. Around 61.8% (55/89) were males and 55.1% (49/89) were obese (BMI≥28 kg/m^2^). Overall, 34.8% (31/89) had hypertension, 70.8% (63/89) had dyslipidemia, and 6.7% (6/89) had type 2 diabetes mellitus. There were no significant differences between the 2 groups in terms of age, sex, weight, presence of comorbidities, biochemical variables, diet, or activity-related parameters (*P*>.05). Additionally, no difference was found in the CAP or LSM between the groups at baseline (*P>.05*).

**Table 1.** Baseline characteristics of the total study population and the randomized allocation groups in intention-to-treat population.

Characteristic	Total (N=89)	Control (n=44)	Intervention (n=45)	*P* value^a^
Sex (male/female), n(%)	55 (61.8)/ 34(38.2)	31 (70.5)/ 13(29.5)	24 (53.3)/ 21(46.7)	.10
Age (years), mean (SD; 95% CI)	40.0 (9.8; 37.9-42.1)	40.7 (10.5; 37.5-43.9)	39.3 (9.1; 36.6-42.1)	.84
Weight (kg), mean (SD; 95% CI)	82.5 (11.6; 80.1-85.0)	82.3 (11.2; 78.9-85.7)	82.7 (12.1; 79.1-86.4	.92
BMI (kg/m^2^), mean (SD; 95% CI)	28.6 (3.0; 27.9-29.2	28.2 (3.0; 27.2-29.1)	29.0 (2.9; 28.1-29.8)	.25
BMI≥28 (kg/m^2^), n (%)	49 (55.1)	21 (47.7)	28 (50.9)	.75
WC^b^ (cm), mean (SD; 95% CI)	92.8 (8.9; 90.9-94.7)	92.6 (8.9; 89.9-95.3)	93.0 (9.0; 90.3-95.7)	.97
Waist to hip ratio, mean (SD; 95% CI)	0.90 (0.06; 0.89-0.92)	0.91 (0.06; 0.89-0.93)	0.90 (0.07; 0.88-0.92)	.38
Waist to height ratio, mean (SD; 95% CI)	0.55 (0.05; 0.54-0.56)	0.54 (0.05; 0.53-0.56)	0.55 (0.04; 0.54-0.56)	.45
Systolic blood pressure (mmHg), mean (SD; 95% CI)	123.8 (6.8; 122.4-125.3)	124.4 (6.7; 122.4-126.5)	123.3 (7.0; 121.2-125.4)	.24
Diastolic blood pressure (mmHg), mean (SD; 95% CI)	79.3 (5.7; 78.1-80.5)	79.6 (5.9; 77.9-81.4)	79.0 (5.6; 77.3-80.7)	.35
Hypertension, n (%)	31 (34.8)	17 (38.6)	14 (31.1)	.46
Type 2 diabetes mellitus, n (%)	6 (6.7)	4 (9.1)	2 (4.4)	.43
Dyslipidemia, n (%)	63 (70.8)	33 (75)	30 (66.7)	.39
Hyperuricemia, n (%)	34 (38.2)	18 (40.9)	16 (35.6)	.60
Metabolic syndrome, n (%)	37 (41.6)	19 (43.2)	18 (40)	.76
Platelet count (10^9^/L), mean (SD; 95% CI)	256.6 (51.7; 245.8-267.5)	254.5 (51.9; 238.7-270.3)	258.8 (51.9; 243.2-274.4)	.58
ALT^c^ (U/L), median (IQR)	36.0 (26.0-59.0)	38.0 (24.5-57.3)	34.0 (26.5-61.0)	.90
AST^d^ (U/L), median (IQR)	26.0 (21.0-36.0)	26.0 (21.3-35.5)	25.0 (19.5-36.0)	.68
GGT^e^ (U/L), median (IQR)	35.0 (23.0-53.5)	39.0 (25.3-59.8)	34.0 (22.0-50.0)	.16
Albumin (g/L), mean (SD; 95% CI)	46.3 (2.4; 45.8-46.8)	46.6 (2.0; 46.0-47.2)	45.9 (2.6; 45.1-46.7)	.20
Urea (mmol/L), mean (SD; 95% CI)	4.9 (1.1; 4.7-5.1)	5.0 (1.1; 4.6-5.3)	4.9 (1.0; 4.6-5.2)	.75
Serum creatinine (μmol/L), mean (SD; 95% CI)	66.8 (14.4; 63.8-70.0)	68.9 (14.0; 64.6-73.1)	64.9 (14.6; 60.5-69.3)	.18
Uric acid (μmol/L), mean (SD; 95% CI)	410.7 (109.9; 387.5-433.8)	416.1 (112.5; 381.9-450.2)	405.4 (108.3; 372.9-437.9)	.62
Fasting plasma glucose (mmol/L), mean (SD; 95% CI)	5.3 (0.6; 5.2-5.5)	5.4 (0.6; 5.2-5.5)	5.3 (0.6; 5.1-5.5)	.54
Fasting insulin (pmol/L), mean (SD; 95% CI)	78.0 (39.3; 69.7-86.2)	80.1 (45.6; 66.2-94.0)	76.0 (32.4; 66.2-85.7)	.93
HOMA-IR^f^, median (IQR)	2.4 (1.7-3.3)	2.4 (1.6-3.4)	2.4 (1.7-3.2)	.96
HOMA-IR≥2.5, n (%)	40 (44.9)	19 (43.2)	21 (46.7)	.74
Hemoglobin A_1c_ (%), median (IQR)	5.6 (5.4-5.8)	5.6 (5.2-5.8)	5.6 (5.4-5.9)	.36
TG^g^ (mmol/L), median (IQR)	1.6 (1.0-2.0)	1.6 (1.0-2.2)	1.5 (1.0-1.8)	.43
TC^h^ (mmol/L), median (IQR)	4.9 (4.2-5.3)	4.9 (4.2-5.4)	5.0 (4.3-5.4)	.78
HDL-C^i^ (mmol/L), median (IQR)	1.2 (1.0-1.3)	1.1 (1.0-1.3)	1.2 (1.0-1.4)	.26
LDL-C^j^ (mmol/L), median (IQR)	3.2 (2.6-3.7)	3.3 (2.6-3.8)	3.2 (2.5-3.6)	.68
CAP^k^ (dB/m), median (IQR)	334 (295-365)	336 (288-362)	334 (297-369)	.25
Liver stiffness measurement (kPa), median (IQR)	5.1 (4.4-6.5)	5.2 (4.4-6.4)	4.9 (4.4-6.7)	.56
Diet quality score, median (IQR)	57 (50-66)	56 (49-66)	58 (51-66)	.64
Total MET^l^-minutes, median (IQR)	1116 (544-2057)	1118 (620-2068)	1102 (465-2088)	.59

a Continuous variables were assessed using unpaired t tests (normally distributed data) and Mann-Whitney *U* tests (non-normally distributed data) between groups. Categorical variables were analyzed using the chi-square test or Fisher exact test between groups.

b WC: waist circumference.

c ALT: alanine aminotransferase.

d AST: aspartate aminotransferase.

e GGT: γ-glutamyl transpeptidase.

f HOMA-IR: homeostasis model assessment-insulin resistance.

g TG: triglyceride.

h TC: total cholesterol.

i HDL-C: high-density lipoprotein-cholesterol.

j LDL-C: low-density lipoprotein-cholesterol.

k CAP: controlled attenuation parameter.

l MET: metabolic equivalent of task.

### Changes in Clinical Parameters

**Figure 2.**
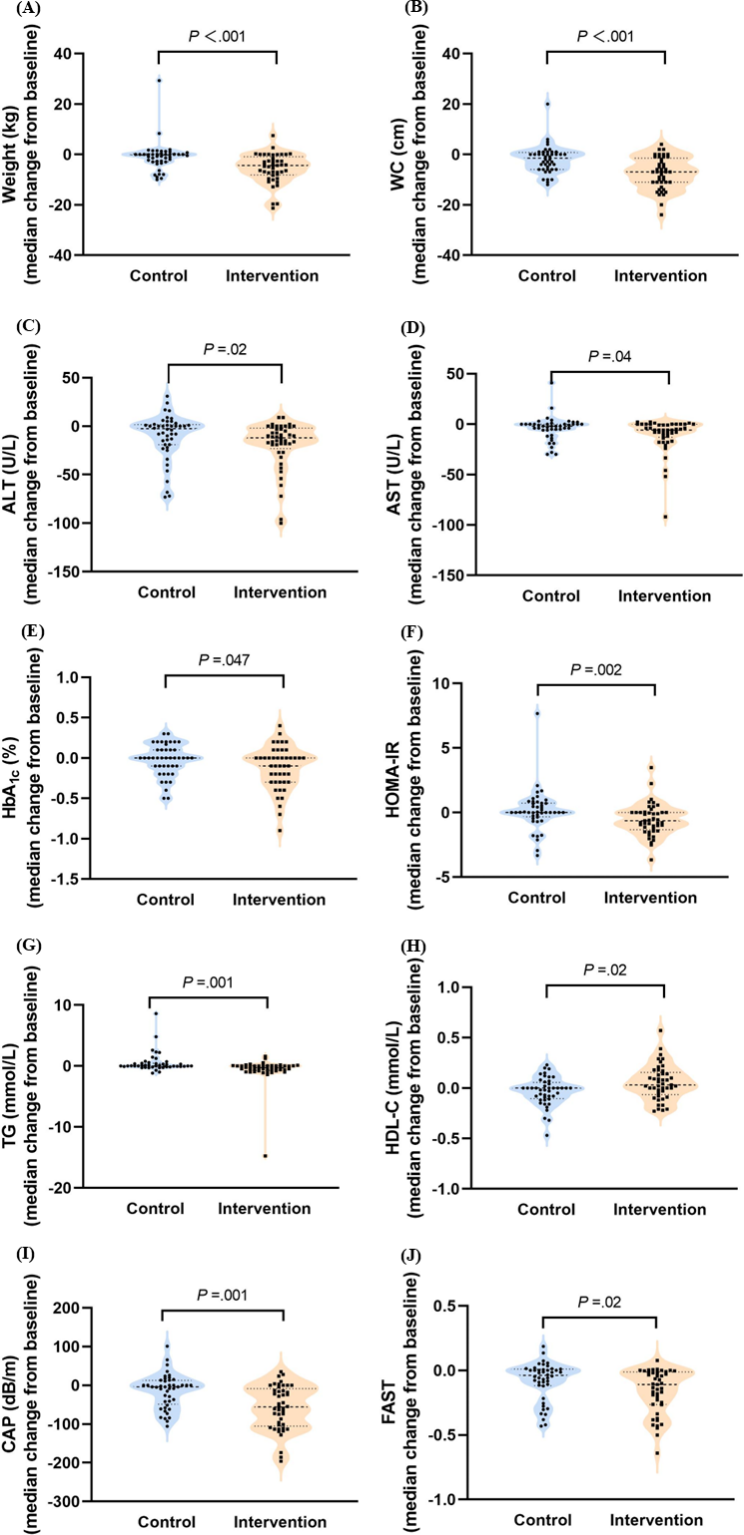
Change in clinical parameters from baseline to 24 weeks between control group and intervention group. (A) Median change in weight between groups (–0.3 vs –4.4; median difference –3.7, 95% CI –5.5 to –1.7; *P*<.001). (B) Median change in waist circumference between groups (–1.5 vs –7.0; median difference –5.0, 95% CI –7.0 to –2.0; *P*<.001). (C) Median change in alanine aminotransferase values between groups (–2.5 vs –12.0; median difference –8.0, 95% CI –15.0 to –1.0; *P*=.02). (D) Median change in aspartate aminotransferase values between groups (–2.0 vs –6.0; median difference –3.0, 95% CI –6.0 to 0; *P*=.04). (E) Median change in hemoglobin A_1c_ between groups (0 vs –0.1; median difference –0.1, 95% CI –0.2 to 0; *P*=.047). (F) Median change in homeostasis model assessment-insulin resistance between groups (0 vs –0.6; median difference –0.7, 95% CI –1.1 to –0.2; *P*=.002). (G) Median change in triglyceride values between groups (0 vs –0.3; median difference –0.5, 95% CI –0.7 to –0.2; *P*=.001). (H) Median change in high-density lipoprotein-cholesterol values between groups (0 vs 0.1; median difference 0.1, 95% CI 0-0.1; *P* =.02). (I) Median change in controlled attenuation parameter values between groups (–4.0 vs –56.0; median difference –38, 95% CI –62 to –16; *P*=.001). (J) Median change in FibroScan-aspartate aminotransferase scores between groups (–0.04 vs –0.11; median difference –0.06, 95% CI –0.13 to –0.01; *P* =.02). ALT: alanine aminotransferase; AST: aspartate aminotransferase; CAP: controlled attenuation parameter; FAST: FibroScan–aspartate aminotransferase; HbA_1c_: hemoglobin A_1c_; HDL-C: high-density lipoprotein cholesterol; HOMA-IR: homeostasis model assessment–insulin resistance; TG: triglyceride; WC: waist circumference.

Table 2 and Figure 2 present the clinical parameters at baseline and 24 weeks and their changes between groups in intention-to-treat population. There was a significant reduction in weight, waist circumference, waist-to-height ratio, and BMI from baseline within each group (*P*<.05). The reductions in these factors were also significantly greater in the intervention group than in the control group (*P*<.05). Patients in the TLC program intervention group had lower alanine aminotransferase (ALT), aspartate aminotransferase (AST), and γ-glutamyl transpeptidase (GGT) levels at 6 months than at baseline, and these liver enzyme levels decreased more in the intervention group than in the control group (*P*<.05). At 6 months, reductions in the levels of glucose metabolism–related factors, including fasting insulin, hemoglobin A_1c_ (HbA_1c_), and HOMA-IR, were observed only within the intervention group (*P*<.05), and the levels of these factors showed significantly greater reductions in the intervention group than in the control group (*P*<.05). However, there was no between-group difference in FPG (*P*=.22). Patients in the intervention group had greater reductions in triglyceride (median difference −0.5, 95% CI −0.7 to −0.2; *P*=.001) and increases in high-density lipoprotein-cholesterol (HDL-C) levels than did those in the control group (median difference 0.1, 95% CI 0‐0.1; *P*=.02). The diet quality score improved more in the TLC program group than in the control group (median difference 6, 95% CI 0‐10; *P*=.04), whereas MET did not significantly differ between the groups (*P*=.92).

**Table 2.** Clinical parameters between the two study groups at baseline and 24 weeks in the intention-to-treat population.

Parameter	Control (n=44)			Intervention (n=45)			Mean or Hodges-Lehmann median difference (95% CI)^a^	*P* value^a^
	Baseline	24 weeks	*P* value^b^	Baseline	24 weeks	*P* value^b^		
Weight (kg), mean (SD; 95% CI)	82.3 (11.2; 78.9-85.7)	81.4 (12.2 ;77.7-85.0)	.02	82.7 (12.1; 79.1-86.4)	77.2 (11.7; 73.7-80.8)	<.001	–3.7 (–5.5 to –1.7)	<.001
BMI (kg/m^2^), mean (SD; 95% CI)	28.2 (3.0; 27.2-29.1)	27.5 (3.4; 26.5-28.6)	.004	29.0 (2.9; 28.1-29.8)	26.9 (2.9; 26.0-27.8)	<.001	–1.4 (–2.1 to –0.7)	<.001
WC^c^(cm), mean (SD; 95% CI)	92.6 (8.9; 89.9-95.3)	90.6 (8.6; 88.0-93.2)	.003	93.0 (9.0; 90.3-95.7)	85.3 (9.5; 82.7-88.4)	<.001	–5.0 (–7.0 to –2.0)	<.001
Waist to hip ratio, mean (SD; 95% CI)	0.91 (0.06; 0.89-0.93)	0.90 (0.06; 0.89-0.92)	.21	0.90 (0.07; 0.88-0.92)	0.87 (0.07; 0.85-0.89)	<.001	–0.02 (–0.04 to 0.01)	.06
Waist to height ratio, mean (SD; 95% CI)	0.54 (0.05; 0.53-0.56)	0.53 (0.05; 0.52-0.55)	.003	0.55 (0.04; 0.54-0.56)	0.50 (0.05; 0.49-0.52)	<.001	–0.03 (–0.04 to –0.02)	<.001
Systolic blood pressure (mmHg), mean (SD; 95% CI)	124.4 (6.7; 122.4-126.5)	125.6 (9.9; 122.6-128.6)	.98	123.3 (7.0; 121.2-125.4)	123.4 (11.3; 120.0-126.8)	.17	–1.0 (–6.0 to 4.0)	.87
Diastolic blood pressure (mmHg), mean (SD; 95% CI)	79.6 (5.9; 77.9-81.4)	81.1 (5.9; 79.3-82.9)	.18	79.0 (5.6; 77.3-80.7)	79.6 (5.8; 77.8-81.3)	.47	–0.9 (–3.7 to 1.9)	.62
Platelet count (10^9^/L), mean (SD; 95% CI)	254.5 (51.9; 238.7-270.3)	256.0 (66.5; 235.7-276.2)	.92	258.8 (51.9; 243.2-274.4)	250.0 (44.3; 237.0-262.0)	.03	–12.8 (–26.7 to 1.1)	.07
ALT^d^ (U/L), median (IQR)	38.0 (24.5-57.3)	30.0 (22.3-44.3)	.01	34.0 (26.5-61.0)	23.0 (17.0-33.5)	<.001	–8.0 (–15.0 to –1.0)	.02
AST^e^ (U/L), median (IQR)	26.0 (21.3-35.5)	22.0 (19.0-27.8)	.003	25.0 (19.5-36.0)	19.0 (15.0-23.0)	<.001	–3.0 (–6.0 to 0)	.04
GGT^f^ (U/L), median (IQR)	39.0 (25.3-59.8)	41.0 (25.3-57.5)	.76	34.0 (22.0-50.0)	23.0 (16.5-34.5)	<.001	–8.0 (–14.0 to –3.0)	<.001
Albumin (g/L), mean (SD; 95% CI)	46.6 (2.0; 46.0-47.2)	45.7 (2.1; 44.8-46.0)	.001	45.9 (2.6; 45.1-46.7)	45.1 (4.0; 43.9-46.3)	.15	0.4 (–0.8 to 1.6)	.06
Urea (mmol/L), mean (SD; 95% CI)	5.0 (1.1; 4.6-5.3)	5.0 (1.2; 4.6-5.4)	.40	4.9 (1.0; 4.6-5.2)	5.0 (1.1; 4.7-5.3)	.61	0.1 (–0.3 to 0.4)	.95
Serum creatinine (μmol/L), mean (SD; 95% CI)	68.9 (14.0; 64.6-73.1)	68.7 (13.8; 64.5-72.9)	.85	64.9 (14.6; 60.5-69.3)	64.5 (14.0; 60.3-68.7)	.78	–0.2 (–2.2 to 1.8)	.79
Uric acid (μmol/L), mean (SD; 95% CI)	416.1 (112.5; 381.9-450.2)	404.1 (75.2; 380.4-427.2)	.57	405.4 (108.3; 372.9-437.9)	386.8 (105.6; 355.0-418.5)	.008	–6.6 (–36.1 to 22.8)	.16
Fasting plasma glucose (mmol/L), mean (SD; 95% CI)	5.4 (0.6; 5.2-5.5)	5.5 (0.9; 5.3-5.8)	.39	5.3 (0.6; 5.1-5.5)	5.3 (0.5; 5.1-5.4)	.24	–0.1 (–0.5 to 0.3)	.22
Fasting insulin (pmol/L), mean (SD; 95% CI)	80.1 (45.6; 66.2-94.0)	81.0 (40.8; 68.6-93.4)	.35	76.0 (32.4; 66.2-85.7)	60.1 (34.8; 49.6-70.6)	.001	–16.8 (–30.0 to –3.5)	.002
HOMA-IR^g^, median (IQR)	2.4 (1.6-3.4)	2.7 (1.9-3.7)	.25	2.4 (1.7-3.2)	1.7 (1.1-2.8)	.001	–0.7 (–1.1 to –0.2)	.002
HbA_1c_^h^ (%), median (IQR)	5.6 (5.2-5.8)	5.5 (5.3-5.7)	.58	5.6 (5.4-5.9)	5.4 (5.3-5.7)	.003	–0.1 (–0.2 to 0)	.047
TG^i^ (mmol/L), median (IQR)	1.6 (1.0-2.2)	1.9 (1.2-2.4)	.09	1.5 (1.0-1.8)	1.0 (0.8-1.5)	.001	–0.5 (–0.7 to –0.2)	.001
TC^j^ (mmol/L), median (IQR)	4.9 (4.2-5.4)	4.6 (4.0-5.2)	.15	5.0 (4.3-5.4)	4.5 (3.9-5.1)	.009	–0.1 (–0.4 to 0.1)	.32
HDL-C^k^ (mmol/L), median (IQR)	1.1 (1.0-1.3)	1.1 (0.9-1.3)	.21	1.2 (1.0-1.4)	1.3 (1.0-1.5)	.07	0.1 (0-0.1)	.02
LDL-C^l^ (mmol/L), median (IQR)	3.3 (2.6-3.8)	3.1 (2.6-3.6)	.06	3.2 (2.5-3.6)	3.1 (2.3-3.5)	.04	–0.1 (–0.2 to 0.1)	.75
CAP^m^ (dB/m), median (IQR)	336 (288-362)	310 (288-342)	.04	334 (297-369)	291 (239-317)	<.001	–38 (–62 to –16)	.001
CAP<268, n (%)	4 (9.1)	6 (13.6)	.55	1 (2.2)	16 (35.6)	<.001	—^n^	—
268≤CAP<280, n (%)	4 (9.1)	3 (6.8)	—	1 (2.2)	4 (8.9)	—	—	—
CAP≥280, n (%)	36 (81.8)	35 (79.5)	—	43 (95.6)	25 (55.6)	—	—	—
Fatty liver index, mean (SD; 95% CI)	58.4 (22.3; 51.6-65.2)	57.1 (22.2; 50.3-63.8)	.38	56.7 (21.8; 50.2-63.3)	34.2 (22.3; 27.5-40.9)	<.001	–21.1 (–28.4 to –13.9)	<.001
Liver stiffness measurement (kPa), median (IQR)	5.2 (4.4-6.4)	5.1 (4.1-6.8)	.07	4.9 (4.4-6.7)	5.1 (4.0-6.0)	.16	–0.1 (–0.6 to 0.5)	.94
AST-to-platelet ratio index, median (IQR)	0.28 (0.20-0.37)	0.24 (0.18-0.30)	.008	0.27 (0.19-0.35)	0.21 (0.16-0.27)	<.001	–0.03 (–0.06 to 0.01)	.13
Fibrosis-4 index, median (IQR)	0.68 (0.50-1.00)	0.66 (0.46-0.99)	.20	0.64 (0.49-0.90)	0.62 (0.49-0.76)	.15	–0.05 (–0.12 to 0.03)	.85
NAFLD^o^ fibrosis score, mean (SD; 95% CI)	–2.9 (1.2; –3.2 to –2.5)	–2.6 (1.2; –3.0 to –2.3)	.09	–2.9 (1.0; –3.2 to –2.6)	–2.7 (1.0; –3.0 to –2.4)	.15	–0.1 (–0.4 to 0.3)	.96
FAST^p^, median (IQR)	0.20 (0.11-0.42)	0.11 (0.07-0.22)	.001	0.22 (0.09-0.43)	0.06 (0.03-0.15)	<.001	–0.06 (–0.13 to –0.01)	.02
Agile3+, median (IQR)	0.06 (0.02-0.15)	0.05 (0.02-0.15)	.96	0.05 (0.02-0.12)	0.05 (0.03-0.12)	.63	0.01 (–0.02 to 0.03)	.68
Diet quality score, median (IQR)	56 (49-66)	64 (55-71)	.01	58 (51-66)	66 (61-76)	<.001	6 (0-10)	.04
Total MET^q^-minutes, median (IQR)	1118 (620-2068)	1442 (620-2608)	.29	1102 (465-2088)	1102 (647-1725)	.44	–10 (–204 to 192)	.92

a Continuous variables were assessed using 2-tailed unpaired *t* tests (reporting mean difference and 95% CI) for normally distributed data and Mann-Whitney *U* tests (reporting Hodges-Lehmann median difference and 95% CI) for non-normally distributed data to compare the changes between the two groups.

b Continuous variables were assessed using paired *t* tests (normally distributed data) or Wilcoxon signed-rank tests (non-normally distributed data) within each group. Categorical variables were analyzed using marginal homogeneity tests within each group.

c WC: waist circumference.

d ALT: alanine aminotransferase.

e AST: aspartate aminotransferase.

f GGT: γ-glutamyl transpeptidase.

g HOMA-IR: homeostasis model assessment-insulin resistance.

h HbA_1c_: hemoglobin A_1c_.

i TG: triglyceride.

j TC: total cholesterol.

k HDL-C: high-density lipoprotein-cholesterol.

l LDL-C: low-density lipoprotein-cholesterol.

m CAP: controlled attenuation parameter.

n Not applicable.

o NAFLD: nonalcoholic fatty liver disease.

p FAST: FibroScan-aspartate aminotransferase.

q MET: metabolic equivalent of task.

### Changes in Noninvasive Factors of Hepatic Steatosis and Liver Fibrosis

At 6 months, both groups presented a reduction in the CAP value. The intervention group experienced a decrease to 291(239-317) dB/m, whereas the control group had a median CAP of 310 (288-342)dB/m (Table 2, *P*=.001). CAP values were significantly lower in the intervention group than in the control group (Figure 2, median difference −38, 95% CI −62 to −16; *P*=.001). In the subgroup analysis, the percentage of patients with severe steatosis decreased significantly in the intervention group from baseline at 6 months (*P*<.001). However, the LSM values were not significantly different between groups or within each group (*P*>.05). Based on the noninvasive assessment, patients in the TLC program intervention group had lower FAST scores than did those in the control group (median difference −0.06, 95% CI −0.13 to −0.01; *P*=.02). In the comparison of these 2 groups, no significant differences were found in noninvasive scores, including the APRI, FIB-4, NFS, and Agile 3+ score (*P*>.05).

### Changes in Body Composition

According to the body composition analysis (Table 3), each group presented a decrease in total body fat mass, total body fat percentage, and visceral fat level, and these variables decreased more in the intervention group than in the control group across the 6-month period (*P*<.05). The basal metabolic rate underwent a greater reduction in the intervention group than in the control group (mean difference −42, 95% CI −62 to −22; *P*<.001). The intervention group also presented more pronounced decreases in fat-free mass, total lean body mass, and appendicular skeletal muscle mass (ASM; *P*<.05). The ratio of ASM to weight increased in the intervention group, but no significant difference was observed in this ratio between the groups (*P*>.05).

**Table 3.** Body composition analysis between the two study groups at baseline and 24 weeks in the intention-to-treat population.

Body composition	Control n=44			Intervention n=45			Mean or Hodges-Lehmann median difference (95% CI)^a^	*P* value^a^
	Baseline	24weeks	*P* value^b^	Baseline	24 weeks	*P* value^b^		
Total body fat mass (kg), mean (SD; 95% CI)	25.3 (6.8; 23.3-27.4)	24.3 (7.0; 22.2-26.4)	.006	28.3 (7.3; 26.2-30.6)	24.6 (7.3; 22.4-26.7)	<.001	–2.8 (–4.4 to –1.2)	<.001
Total body fat percentage (%), mean (SD; 95% CI)	30.7 (6.9; 28.6-32.8)	29.9 (6.8; 27.9-32.0)	.002	34.3 (7.8; 32.0-36.7)	30.9 (8.5; 28.3-33.5)	<.001	–2.6 (–4.1 to –1.2)	<.001
Visceral fat level, median (IQR)	13 (11-16)	12 (10-14)	.001	12 (10-15)	10 (8-13)	<.001	–1 (–2 to 0)	<.001
Fat-free mass (kg), mean (SD; 95% CI)	57.0 (9.1; 54.2-59.7)	57.1 (9.7; 54.1-60.0)	.44	54.3 (10.6; 51.1-57.5)	52.7 (10.1; 49.6-55.7)	<.001	–1.1 (–1.9 to –0.2)	.01
Total lean body mass (kg), mean (SD; 95% CI)	53.4 (9.2; 50.6-56.1)	52.7 (9.3; 49.9-55.5)	.29	51.0 (10.0; 48.0-54.0)	49.9 (9.9; 47.0-52.9)	<.001	–0.7 (–1.2 to –0.2)	.002
ASM^c^ (kg), mean (SD; 95% CI)	25.5 (5.4; 23.8-27.1)	25.0 (5.8; 23.2-26.8)	.70	24.8 (5.9; 23.0-26.6)	23.9 (5.7; 22.2-25.6)	<.001	–0.6 (–1.0 to –0.3)	.001
ASM to weight ratio (%), mean (SD; 95% CI)	30.7 (3.8; 29.6-31.9)	30.8 (5.3; 29.1-32.3)	.009	29.8 (4.4; 28.5-31.1)	30.8 (4.5; 29.5-32.1)	<.001	0.5 (–0.1 to 1.1)	.08
Trunk muscle mass (kg), mean (SD; 95% CI)	27.9 (4.1; 26.6-29.1)	27.7 (4.1; 26.5-29.0)	.31	26.2 (4.5; 24.9-27.6)	26.0 (4.4; 24.7-27.3)	.06	–0.1 (–0.5 to 0.1)	.27
Total body water (kg), mean (SD; 95% CI)	38.1 (5.3; 36.5-39.7)	38.2 (5.4; 36.6-39.8)	.54	37.7 (5.6; 36.1-39.5)	36.9 (5.6; 35.2-38.6)	.001	–0.9 (–1.5 to –0.4)	.003
Total body water percentage (%), mean (SD; 95% CI)	47.0 (3.9; 45.8-48.1)	47.1 (5.1; 45.5-48.6)	.07	46.0 (4.0; 44.9-47.3)	47.8 (5.2; 46.3-49.4)	<.001	1.6 (0.2-3.0)	.001
Extracellular water percentage (%), mean (SD; 95% CI)	41.2 (2.1; 40.5-41.8)	40.9 (2.0; 40.3-41.5)	.003	41.4 (2.2; 40.7-42.0)	40.8 (2.0; 40.2-41.4)	.001	–0.2 (–0.6 to 0.1)	.10
BMC^d^ (kcal), mean (SD; 95% CI)	1631 (252; 1555-1708)	1621 (251; 1545-1698)	.27	1597 (273; 1515-1679)	1545 (274; 1463-1627)	<.001	–42 (–62 to –22)	<.001

a Continuous variables were assessed using unpaired *t* tests (reporting mean difference and 95% CI) for normally distributed data and Mann-Whitney *U* tests (reporting Hodges-Lehmann median difference and 95% CI) for non-normally distributed data to compare the changes between the two groups.

b Continuous variables were assessed using paired *t* tests (normally distributed data) or Wilcoxon signed-rank tests (non-normally distributed data) within each group.

c ASM: appendicular skeletal muscle mass.

d BMC: basal metabolic rate.

### Analysis of Primary and Secondary Efficacy Outcomes

**Figure 3.**
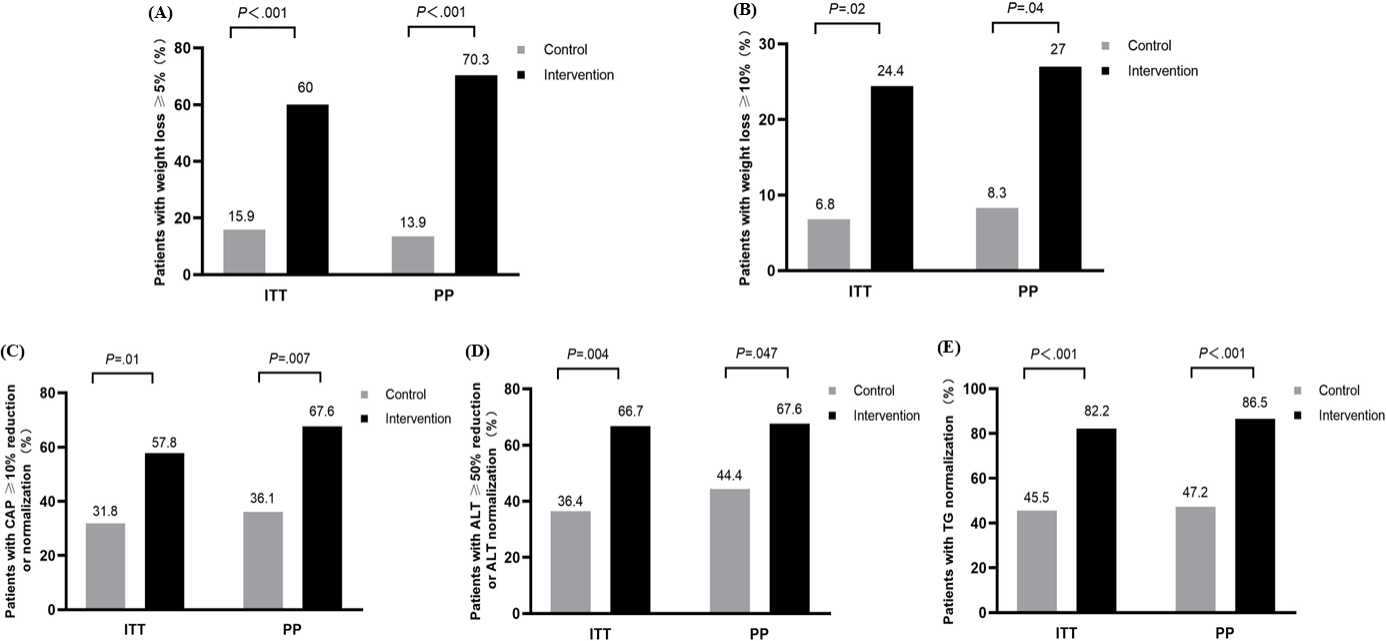
Analysis of primary and secondary efficacy outcome between control group and intervention group. (A) The percentage of patients with weight loss ≥5% between groups in the intention-to-treat population (27/45 vs 7/44, RR 3.771, 95% CI 1.836-7.748; *P*<.001 ) and the per-protocol population (26/37 vs 5/36, RR 5.059, 95% CI 2.184-11.719; *P* <.001) ; (B) The percentage of patients with weight loss ≥10% between groups in the intention-to-treat population (11/45 vs 3/44, RR 3.585, 95% CI 1.072-11.988; *P*=.02) and the per-protocol population (10/37 vs 3/36, RR 3.243, 95% CI 1.066-10.360; *P*=.04) ; (C) The percentage of patients with controlled attenuation parameter ≥10% reduction or normalization between groups in the intention-to-treat population (26/45 vs 14/44, RR 1.816, 95% CI 1.102-2.992; *P=*.01 ) and the per-protocol population (25/37 vs 13/36, RR 1.871, 95% CI: 1.148-3.050; *P*=.007; (D) The percentage of patients with alanine aminotransferase ≥50% reduction or normalization between groups in the intention-to-treat population (30/45 vs 16/44, RR 1.833, 95% CI 1.178-2.853; *P*=.004 ) and the per-protocol population (25/37 vs 16/36, RR 1.520, 95% CI 1.006-2.391; *P*=.047; (E) The percentage of patients with triglyceride normalization between groups in the intention-to-treat population (37/45 vs 20/44, RR 1.809, 95% CI 1.273-2.570; *P*<.001 ) and the per-protocol population (32/37 vs 17/36, RR 1.831, 95% CI 1.267-2.646; *P*<.001). ALT: aspartate aminotransferase; CAP: controlled attenuation parameter; ITT: intention-to-treat; PP: per-protocol; TG: triglyceride.

As shown in Figure 3, the primary and secondary efficacy outcomes were compared between the control and intervention groups. According to the ITT analysis Figure 3, 60% (27/45) of patients achieved weight loss of ≥5% (27/45 vs 7/44; RR 3.771, 95% CI 1.836‐7.748; *P*<.001), and 24.4 % (11/45) of patients attained weight loss of ≥10% among patients in the TLC program-delivered intervention group, which was greater than those in the control group (11/45 vs 3/44; RR 3.585, 95% CI 1.072‐11.988; *P*=.02). A CAP value of ≥10% reduction or normalization was achieved in more patients who received the intervention than in those who received standard care (26/45 vs 14/44; RR 1.816, 95% CI 1.102‐2.992; *P*=.01). Furthermore, patients in the intervention group were more likely than those in the control group to achieve an ALT reduction of ≥50% or normalization (30/45 vs 16/44; RR 1.833, 95% CI 1.178‐2.853; *P*=.004), as well as triglyceride normalization (37/45 vs 20/44; RR 1.809, 95% CI 1.273‐2.570; *P*<.001). Similarly, these results from the PP analysis were in line with those from the ITT analysis (Figure 3).

The effects of the TLC program on weight loss and the CAP value were also analyzed by multivariate regression (Table 4). Among the ITT population or PP population, the intervention had a positive effect on weight loss of ≥5% or ≥10% after adjusting for age, sex, and baseline weight (*P*<.05). Additionally, patients in the intervention group were more likely to achieve a CAP reduction of ≥10% or normalization after adjusting for age, sex, and baseline CAP in both the ITT population (OR 2.853, 95% CI 1.092‐7.456; *P*=.03) and the PP population (OR 3.319, 95% CI 1.117‐9.860; *P*=.03).

**Table 4.** Multivariate logistic analysis model of reduction in weight and controlled attenuation parameter value in intention-to-treat and per-protocol population.

Parameter	Unadjusted	Model 1^a^	Model 2^b^	Unadjusted	Model 1	Model 2
	ITT^c^ population (N=89)			PP^d^ population (N=73)		
Weight loss≥5%						
Control	reference	reference	reference	reference	reference	reference
Intervention, OR (95% CI)	7.929 (2.905-21.641)	8.537 (2.947-24.730)	8.380 (2.886-24.331)	14.655 (4.509-47.626)	14.534 (4.267-49.499)	15.231 (4.380-52.969)
*P* value	＜.001	＜.001	＜.001	＜.001	＜.001	＜.001
Weight loss ≥10%						
Control	reference	reference	reference	reference	reference	reference
Intervention, OR^e^ (95% CI)	4.422 (1.140‐17.144)	4.677 (1.160‐18.855)	4.612 (1.138‐18.686)	4.074 (1.018‐16.305)	4.604 (1.093‐19.386)	4.571 (1.078‐19.387)
*P* value	.03	.03	.03	.047	.04	.04
CAP^f^≥10% reduction or normalization						
Control	reference	reference	reference	reference	reference	reference
Intervention, OR (95% CI)	2.932 (1.232-6.981)	2.843 (1.161-6.965)	2.853 (1.092-7.456)	3.686 (1.401-9.700)	3.399 (1.259-9.175)	3.319 (1.117-9.860)
*P* value	.02	.02	.03	.008	.02	.03

a Model 1: Adjusted for age, sex.

b Model 2: Adjusted for model 1 and baseline weight.

c ITT: intention-to-treat.

d PP: per-protocol population.

e OR: odds ratio.

f Model 2：Adjusted for model 1 and baseline controlled attenuation parameter value; CAP: controlled attenuation parameter.

### Safety

During the study period, the intervention was safe in this cohort of patients with MAFLD, with no adverse events reported during the study period. The heart rate monitoring during the video exercises effectively protected participants from the risk of overexertion.

## Discussion

### Principal Findings

MAFLD is a public health concern and is associated with unhealthy lifestyles. Effective weight control is a crucial measure for reversing MASH and attenuating metabolic dysfunction [11,33]. While digital health tools offer a scalable solution, there is a notable scarcity of interventions specifically designed for the population of individuals with MAFLD in China. Therefore, we conducted a longitudinal study to evaluate an innovative WeChat mini-program-based lifestyle modification in patients with MAFLD. Our study revealed that the TLC program intervention was more effective for weight loss among patients with MAFLD who were overweight or obese than standard care was. Importantly, CAP values decreased significantly more in the intervention group than in the control group, suggesting that the TLC program intervention ameliorated hepatic steatosis. Along with weight reduction, the intervention group presented significant decreases in liver enzymes, HbA_1c_, and triglyceride levels, indicating improved liver function and metabolic-related indicators. To investigate the changes in body composition, we found that the intervention group had a lower total body fat percentage and visceral fat level than did the control group, with parallel improvement in hepatic steatosis. In terms of liver fibrosis assessment, only the FAST score showed improvement in MASH with significant fibrosis after the intervention, whereas other fibrosis indicators, such as LSM and noninvasive scores (APRI, FIB-4, NFS, Agile 3+) showed no treatment effect.

Based on established evidence, DTx lifestyle interventions lead to significant weight loss in patients with MAFLD [6,30]. According to a meta-analysis of 8 cohort studies on DTx, 33% of patients with MAFLD experienced significant weight loss of ≥5% [13]. In our study, more than half (27/45, 60%) of the patients with MAFLD achieved a weight loss of ≥5% , demonstrating the efficacy of weight management over 6 months. The significant improvement in diet quality scores observed in the intervention group implies a potential key role of dietary modification within the TLC program. With the reduction in body weight, there was a significant improvement in liver chemistry variables, including ALT, AST, and GGT, which was consistent with published data [30,34]. In particular, approximately two-thirds of the patients with TLC intervention experienced an ALT reduction of more than 50% or normalization, suggesting that this digital intervention improved liver function remarkably.

Importantly, 57.8% (26/45) of the patients in the intervention group presented a CAP value of more than a 10% reduction or normalization, indicating that the severity of hepatic steatosis was highly reduced among individuals after intervention. A similar study investigated a mobile technology–based intervention program for patients with MAFLD and demonstrated that 42.4% (14/33) of participants exhibited a reduction in CAP values, with 21.2% (7/33) achieving a significant decrease of ≥10% in the CAP value [35]. Based on our study, the reduction in body weight and visceral fat led to a marked decline in hepatic fat content and subsequent improvement in liver function.

Among patients with MAFLD, the severity of fibrosis is a major disease modifier and affects hepatic outcomes [36,37]. Gradual weight loss of more than 10% may ameliorate fibrosis in patients with MAFLD [38]. In our study, although the proportion of patients with weight loss greater than 10% reached 24.4% after intervention, the LSM value was not significantly different between the groups. Similarly, no significant difference was found in noninvasive scores, such as the APRI, FIB-4 score, NFS, and Agile 3+ score, except for the FAST score. With respect to noninvasive assessment of liver fibrosis, current evidence from different studies has shown inconsistencies. A German web-based exercise program intervention for patients with MAFLD reported improved APRI, FIB-4 score, and LSM, whereas a US mobile-based program intervention for patients with biopsy-confirmed MAFLD showed no changes in NFS or FIB-4 score [39,40]. The observed discrepancies across studies may stem from variations in intervention protocols, differential durations of implementation, and heterogeneity in patient adherence levels. Based on our data, the overall baseline LSM values of the patients enrolled seem to be remarkably low, with a median value of only 5.1 (IQR 4.4- 6.5) kPa. Furthermore, the duration of the intervention was only 6 months, which is too short for observing changes in fibrosis.

FAST scores reflect MASH with significant fibrosis and are calculated via the CAP, LSM, and AST. In our study, the observed reduction in the FAST score in the intervention group may be associated with the significant decreases in CAP and AST levels, suggesting potential alleviation of MASH to some extent by using the TLC program. Undoubtedly, the definitive evidence of MASH alleviation is contingent upon further study with patients who have undergone liver biopsies.

MAFLD results in impaired metabolic profiles in multiple organ systems, which not only promotes the progression of liver disease but also increases the risk of extrahepatic complications [41,42]. In the current investigation, we assessed key metrics of glucose metabolism, observing substantially greater reductions in fasting insulin, HbA_1c_, and HOMA-IR levels among intervention group participants, which aligns with established findings in the literature [35,39]. Nevertheless, there was no significant difference in the FPG level between these two groups. It is important to emphasize that our cohort consisted predominantly of nondiabetic individuals, with an overall low baseline glucose (5.3 mmol/L), which limited the potential for a large absolute reduction. Interestingly, some studies concerning DTx-mediated lifestyle modifications in MAFLD management have reported similar results [34,40]. Indeed, insulin resistance is the key pathogenic link between obesity and associated metabolic disorders. Moreover, it is the primary driver in the development of MAFLD and cardiovascular disease. A significant reduction in body weight has favorable effects on increasing insulin sensitivity and improving insulin resistance, leading to a reduction in fasting insulin and glycemic fluctuations, substantially reducing cardiovascular events in patients with MAFLD [43]. A reduction in the HbA_1c_ level, reflecting good glycemic control over the past 2‐3 months, may lead to decreased hepatic fat accumulation, reduced liver fat synthesis, and increased fat breakdown [44].

For the lipid profile, decreased TG or elevated HDL-C levels were found in the intervention group, which was in line with previously published literature [35,45,46]. Favorable modifications in TG or HDL profiles have been correlated with the amelioration of insulin resistance, which not only upregulates lipoprotein lipase expression and accelerates TG hydrolysis but also downregulates hepatic lipase activity, reduces HDL-C degradation, and promotes HDL-C synthesis [41,47]. Overall, TLC digital intervention markedly improved metabolic indices, such as glucose and lipid levels, which are pivotal determinants of cardiovascular morbidity and mortality in patients with MAFLD.

To evaluate the quality of weight loss, we explored parameters involved in body composition. Compared with the control group, the intervention group presented greater reductions in total body fat percentage and visceral fat, as well as the alleviation of hepatic steatosis. Although the intervention group demonstrated significant reductions in total lean body mass and absolute ASM, the ratio of ASM to weight increased within the intervention group, suggesting that weight loss was driven primarily by fat reduction. As is well known, healthy weight loss involves fat loss with muscle gain. Furthermore, no significant difference in MET levels was detected between the groups in our study. To build muscle and improve body composition, future improvements to the TLC program should integrate progressive strength training, such as bodyweight exercises, resistance bands, and dumbbells. Monitoring changes in muscle mass using body fat scales can further support the personalization and optimization of these interventions.

### Strengths and Limitations

This study has several strengths. Its innovation lies in being the first tailored digital tool to address the specific self-management challenges of this population in China, leveraging a widely accessible platform. Additionally, this mobile-based program offers customized dietary plans based on each patient’s health status. Notably, this smartphone-delivered lifestyle program aids in weight control and liver fat reduction by facilitating healthier behaviors, which could be implemented in home settings. This high accessibility removes common barriers to care, such as transportation and time constraints, while its timely, personalized feedback promotes greater adherence and sustained habit formation. Together, these advantages make it a scalable and cost-effective strategy for the long-term management of MAFLD.

This study has several limitations. First, all the patients enrolled were from a single tertiary hepatology clinic, and more motivated individuals tended to self-select into the program. This selection bias was inevitable. Second, hepatic steatosis and fibrosis were evaluated by FibroScan or noninvasive tests rather than liver biopsy. On the one hand, FibroScan is a widely used and noninvasive tool to detect hepatic steatosis and screen for fibrosis. On the other hand, it is extremely challenging to obtain liver biopsy specimens in clinical practice. Third, no postintervention follow-up was conducted to assess the maintenance of weight loss. The lack of long-term follow-up data limits the conclusions regarding the sustainability of the WeChat-based intervention effects. Future research will include a follow-up phase where the research team will formulate personalized weight-management plans and encourage the involvement of family or friends for support and supervision to facilitate long-term weight maintenance for participants. Additionally, the sample size herein was limited, and the dropout rate was relatively high (18%). The application of last observation carried forward for handling missing data may have introduced bias into the treatment effect estimate. These factors, collectively, may have contributed to the imprecision in our effect estimates, as evidenced by the wide CIs. Subsequent study should incorporate larger, multicenter cohorts and advanced modeling approaches. Furthermore, the mini-program itself will be further developed to integrate incentives, gamification, and social support. This comprehensive strategy aims to enhance user adherence, boost engagement, and reduce dropout rates.

### Conclusions

Our study introduces a WeChat mini-program that delivers a tailored lifestyle intervention designed for patients with MAFLD in China, addressing a gap in scalable management. Our intervention is characterized by its ability to not only reduce weight but also to significantly improve hepatic steatosis and metabolic measures. These findings contribute to the field by establishing a feasible, low-cost model for managing MAFLD in high-burden populations, with strong potential for implementation in primary care and resource-limited settings. Given the imprecision of effect estimates, further larger-scale studies are essential to confirm these preliminary findings, obtain more precise estimates, and evaluate sustained long-term clinical outcomes with long-term follow-up.

## Supplementary material

10.2196/76204 Multimedia Appendix 1 Institutional Review Board approval document.

10.2196/76204 Checklist 1 CONSORT eHealth checklist (V1.6.1).

## Abbreviations

ALT: alanine aminotransferase
APRI: aspartate aminotransferase-to-platelet ratio index
ASM: appendicular skeletal muscle mass
AST: aspartate aminotransferase
CAP: controlled attenuation parameter
CONSORT: Consolidated Standards of Reporting Trials
DTx: digital therapeutics
FAST: FibroScan-aspartate aminotransferase
FIB-4: fibrosis-4
FPG: fasting plasma glucose
GGT: γ-glutamyl transpeptidase
HbA_1c_: hemoglobin A_1c_
HDL-C: high-density lipoprotein-cholesterol
HOMA-IR: homeostasis model assessment-insulin resistance
IPAQ-SF: International Physical Activity Questionnaire-Short Form
ITT: intention-to-treat
LSM: liver stiffness measurement
MAFLD: metabolic dysfunction–associated fatty liver disease
MASH: metabolic dysfunction-associated steatohepatitis
MET: metabolic equivalent of task
OR: odds ratio
PP: per-protocol
RR: relative risk
TLC: therapeutic lifestyle changes
