# Supplementary material for: Therapeutic Effects of a WeChat Mini-Program on Metabolic Dysfunction–Associated Fatty Liver Disease: Randomized Controlled Trial
Source: J Med Internet Res. 2026 Jan 27;28:e76204. doi: 10.2196/76204 (PMC12843888; doi:10.2196/76204)
Supplement: Multimedia Appendix 1 [file jmir-v28-e76204-s001.pdf]

Approval Letter

Ethics Committee of Xinhua Hospital Affiliated to Shanghai Jiaotong University School of Medicine

Approval Number: XHEC-C-2021-076-2 **Meeting No./Date: M2021-011/2021-09-14**

**Protocol Title/Number**	Application of lifestyle intervention-based digital therapeutics in patients with non-alcoholic fatty liver disease /DTx-NAFLD-1.0
**Reviewed documents**	I. **Initial Review** 1.Application for Ethical Review of Clinical Study; 2. Study Protocol (Version 1.0/2021.07.28); 3. Informed Consent Form (Version 1.0/2021.07.28); 4.Curriculum Vitae of the Principal Investigator; 5. Business License of Hangzhou Jianhai Technology Co., Ltd.<br>II. **Re-review** 1. Application for Re-review; 2. List of Revisions; 3. Study Protocol (Version 2.0/2021.11.23); 4. Informed Consent Form (Version 2.0/2021.11.23)
**CFDA No.**	NA
**Study Product**	NA
**Phase of study**	☐ Phase I Clinical Trial ☐ Phase II Clinical Trial ☐ Phase III Clinical Trial ☐ Phase IV Clinical Trial ☐ Medical Device Registration Trial ☐ Post-Market Medical Device Study ☐ Diagnostic Reagent Registration Trial ☐ Post-Marketing Clinical Study ■ Investigator-Initiated Clinical Research ☐ Other (Please Specify) _____
**Type of review**	■Full Board Review (Initial Review)■ Expedited Review (Re-Review) Emergency Full Board Review
**Study site /PI**	Xinhua Hospital Affiliated to Shanghai Jiaotong University School of Medicine Department of Gastroenterology/Jian-Gao Fan
**Sponsor**	Collaborator: Hangzhou Jianhai Technology Co., Ltd.

Recommendation

- The Ethics Committee conducted the initial review of the project on September 14, 2021. For this meeting, 19 members were expected to attend, 13 were present, and 0 recused themselves. Voting result: Approved with necessary revisions; review opinion: XHEC-C-2021-076-1.
- The Ethics Committee received the re-review application and documents on November 25, 2021. Review result: Approved. This clinical trial must be strictly conducted in accordance with the approved research protocol and informed consent form. Follow-up review frequency: ☐3 months ☐6 months ■12 months ☐Not applicable.

Chairperson (Signature)

Ethics Committee of Xinhua Hospital Affiliated to Shanghai Jiaotong University School of Medicine

December 2, 2021

Notes (Please read carefully):

1. Approved projects must be implemented in accordance with the protocol approved by this Ethics Committee, and shall comply with relevant laws and regulations such as the NMPA and NHC's "Good Clinical Practice for Drugs" (2020), "Good Clinical Practice for Medical Devices" (2016), the NHFPC's "Ethical Review Measures for Biomedical Research Involving Humans" (2016), the WMA's "Declaration of Helsinki" (2013), the CIOMS/WHO's "International Ethical Guidelines for Health-related Research Involving Humans" (2016), and ICH-GCP.
2. This clinical trial should be initiated within one year from the date of approval by the Ethics Committee. If implementation has not commenced by the deadline, this approval document will become automatically void.
3. Any amendments to the research protocol, informed consent form, and other relevant documents during the research process must be submitted to the Ethics Committee for review.
4. To eliminate immediate hazards to subjects, if investigators modify or deviate from the trial protocol without prior Ethics Committee approval, they must promptly report to the Ethics Committee and the sponsor, providing justification, and report to the drug regulatory department when necessary.
5. Upon receiving relevant safety information for the clinical trial provided by the sponsor, the investigator should promptly acknowledge, review it, and consider adjustments to the subject's treatment. If necessary, communicate with the subject as early as possible. Suspected Unexpected Serious Adverse Reactions (SUSARs) provided by the sponsor must be reported to the Ethics Committee.
6. If the sponsor terminates or suspends the clinical trial, the investigator must immediately report this to the clinical trial institution and the Ethics Committee, providing a detailed written explanation.
7. If the Ethics Committee terminates or suspends an approved clinical trial, the investigator must immediately report this to the clinical trial institution and the sponsor, providing a detailed written explanation.
8. The investigator must submit an annual progress report of the clinical trial to the Ethics Committee.
9. If situations arise that may significantly affect the conduct of the trial or increase subject risk, the investigator should provide a written report to the sponsor, the Ethics Committee, and the clinical trial institution as soon as possible.
10. Upon completion of the clinical trial, the investigator must provide the Ethics Committee with a summary of the trial results.

Address:No.1665 Kongjiang Road Shanghai, P.R China,Postcode: 200092 Tel: +86-21-25076141 Fax: +86-21-25078922

伦理委员会会议签到及声明

会议时间：2021 年 9 月 14 日（周二）13:30-16:00　　会议地点：科教大楼 4 楼会议室

姓名	性别	职务	单位	专业	签名	日期
李劲松	男	主任委员	新华医院	核医学科		
陈颖伟	女	副主任委员	新华医院	消化内科	陈颖伟	2021.9.14
陶　荣	男	副主任委员	新华医院	血液科	陶荣	2021.9.14
朱建幸	男	委员	新华医院	新生儿科	[illegible]	2021.9.14
刘振国	男	委员	新华医院	神经内科	[illegible]	2021.9.14
是俊凤	女	委员	新华医院	肾脏内科		
陈　磊	男	委员	新华医院	普外科	陈磊	2021.9.14
顾学范	男	委员	上海市儿科医学研究所	儿科(内分泌遗传代谢)		
齐　隽	男	委员	新华医院	泌尿外科	齐隽	2021.9.14
苏　青	男	委员	新华医院	内分泌科	[illegible]	2021.9.14
陆晓彤	女	委员	新华医院	药学	[illegible]	2021.9.14
蒋更如	男	委员	新华医院	肾脏内科	[illegible]	2021.9.14
张　琦	女	委员	新华医院	眼科	张琦	2021.9.14
沈　伟	女	委员	新华医院	肿瘤科		
袁晓军	女	委员	新华医院	小儿肿瘤	[illegible]	2021.9.14
童剑云	男	委员	上海康正律师事务所	律师	童剑云	2021.9.14
黄承瑶	女	委员	上海康正律师事务所	律师		
范　军	女	委员	上海开放大学	社区代表		
施　敏	男	委员/秘书	新华医院	管理	[illegible]	2021.9.14
黄琦程	女	秘书	新华医院	管理	黄琦程	2021.9.14

声明

- 上海交通大学医学院附属新华医院医学伦理委员会组成及工作程序遵循 ICH-GCP 及中国相关法律法规。
- 伦理委员会已签署同意信守机密或商业专有秘密，并同意信息只以伦理审查为目的，不用于其他目的或公开给第三方。
- 提供审查用的书面资料不能被复制或保留。所有标准操作规程文件、机密信息、摘记及其副本的所有权均归伦理委员会。

Ethics Review Opinion

Ethics Committee of Xinhua Hospital Affiliated to Shanghai Jiao Tong University School of Medicine

XHEC-C-2021-076-1

To: Respectable Investigator, Director Jian-Gao Fan

Regarding the clinical study you submitted (ID: XHEC-C-2021-076), titled " Application of lifestyle intervention-based digital therapeutics in patients with non-alcoholic fatty liver disease," which was reviewed by the Ethics Committee of Xinhua Hospital on September 14, 2021, the voting result is: Approved with Necessary Revisions. The comments are as follows:

1. In the "1. Why is this study being conducted?" section of the informed consent form, include an explanation of what "digital therapy" is.
2. The phrasing "completed the Phase I clinical trial of the corresponding software" in two places in the informed consent form is non-standard. "Registration trial of the software" is more accurate.
3. In the "4. What does this study involve? How long will this study last?" section of the informed consent form, details should be added based on the study protocol regarding how the control (traditional medical treatment) group and the intervention (digital therapy) group will respectively proceed.
4. In the "6. What are the benefits of participating in this study?" section of the informed consent form, add the statement: "You may not receive direct benefit from participating in this study."
5. In the "10. Handling of study-related injuries" section of the informed consent form, the statement "This study will provide insurance for all participating subjects" is recommended to be rephrased as: "The study sponsor has purchased clinical trial liability insurance. If study-related injury occurs, the investigator will bear the associated diagnostic and treatment costs, as well as corresponding financial compensation, in accordance with national laws and regulations." Confirmation is required whether the study sponsor has already purchased clinical trial liability insurance for this study.
6. Are the peripheral devices (Huawei Band 6) provided by the investigator? Will they be collected after the study ends? Regarding the automatic uploading of subject information, clarify where the uploaded information will be stored (which provider's servers), the data retention period, etc.

Please note: This ethics review opinion does not serve as formal approval. Please revise or supplement the materials according to the above comments and resubmit them to the Ethics Committee for review. The study may only commence after formal approval has been obtained. If you have differing views regarding the review opinions, you may provide a written explanation or clarification to this Ethics Committee.

For resubmission, please submit the following materials:

( ) 1. Application for re-review
( ) 2. Submission document checklist
( ) 3. List of revisions
( ) 4. Revised study protocol (with new version number), with modifications highlighted
( ) 5. Revised informed consent form (with new version number), with modifications highlighted
( ) 6. Other requested supplementary documents

Chairperson: [signature]

Ethics Committee of Xinhua Hospital Affiliated to
Shanghai Jiao Tong University School of Medicine (Seal)
September 14, 2021

上海交通大学医学院附属新华医院医学伦理委员会

Cc: Study Sponsor: Hangzhou Jianhai Technology Co., Ltd.

Address:No. 1665 Kongjiang Road Shanghai, P.R China, Postcode:200092 Tel:+86-021-25076143 Fax:+86-021-25078922
